# Ectoparasite and blood parasite prevalence in birds: a comparative study between urbanized airport environments and natural habitats

**DOI:** 10.1007/s00442-025-05811-3

**Published:** 2025-10-11

**Authors:** Victor Aguiar de Souza Penha, Graziela Tolesano-Pascoli, Renata D. Alquezar, Francisco C. Ferreira, Daniela de Angeli Dutra, Érika Martins Braga, Regina H. Macedo, Diego Gil

**Affiliations:** 1 Department of Integrative Biology, Natural Science Building, Michigan State University, 288 Farm Ln, East Lansing, MI 48824 USA; 2 Departamento de Zoologia, Instituto de Ciências Biológicas, Universidade de Brasília, Distrito Federal, Brasília, Brazil; 3 Department of Entomology, Texas A&M University, College Station, TX USA; 4 Department of Biology, Stanford University, Stanford, USA; 5 Departamento de Parasitologia, Universidade Federal de Minas Gerais, Belo Horizonte, Minas Gerais Brazil; 6 Department of Evolutionary Ecology, Museo de Ciencias Naturales (CSIC), Madrid, Spain

**Keywords:** Bayesian multilevel models, Haemosporidian parasites, Ectoparasites, Corticosterone, Body condition

## Abstract

The rapid growth of urban populations and air travel has intensified urbanization’s ecological impacts, reshaping ecosystems through habitat fragmentation, pollution, and species filtering. These effects are particularly evident in urban bird populations. In this study, we examined how urban environments, especially near airports, affect birds by evaluating relationships among parasites, feather corticosterone levels, and body condition. We hypothesized that birds near airports would experience higher parasite prevalence due to stress-related immune compromise. We sampled 1,096 birds from 100 species, recording haemosporidian parasite lineages and ectoparasites across three Brazilian airports, Brasília, Salvador, and Campinas, and corresponding quiet-control areas located 8–17 km away. Using Bayesian multilevel models, we accounted for species relatedness and environmental factors. Overall, 21.8% of birds were infected with haemosporidian parasites. *Turdus leucomelas* showed the highest infection rate (44%), with *Plasmodium* representing 63% of all infections. Ectoparasites infested 9.2% of birds, especially *Elaenia chiriquensis*. Fifteen individuals, mostly in Salvador and Campinas control sites, harbored both parasite groups. Contrary to expectations, haemosporidian prevalence was lower in airport areas than in control sites, while ectoparasites were less common in Brasília’s control site. Feather corticosterone and body condition were not significantly associated with haemosporidian presence, but higher corticosterone correlated with ectoparasite occurrence in control areas. Our results indicate that bird populations around airports exhibit reduced parasite prevalence, suggesting that highly modified environments alter parasite–host dynamics and shape parasite biodiversity. Future studies incorporating infection intensity and longitudinal sampling would help clarify whether urban settings suppress or restructure avian parasite communities.

**Supplementary Information:**

The online version contains supplementary material available at 10.1007/s00442-025-05811-3.

## Introduction

The number of people living in cities has experienced a massive increase in the last fifty years, leading to the expansion of urban areas across all continents (United Nations -– Department of Economic and Social Affairs -– Population Division 2019). As urbanization accelerates globally, so does transportation, with air traffic witnessing a dramatic four-fold increase in passenger numbers over the past three decades (Ritchie and Roser 2021). Airports, as critical nodes in this expanding transportation network, represent highly modified environments that concentrate infrastructure, pollution, noise, and human movement. These hubs intensify the ecological impacts of anthropogenic development by altering habitat structure and facilitating the movement of people, goods, and organisms across regions (Xiong et al. 2022).

Airports and their surrounding areas present distinct ecological conditions compared to rural or natural landscapes (McDonnell et al. 1997), with limited vegetation cover, high levels of disturbance, and restricted wildlife access. Such conditions, which are similar to those found in urban habitats, can reshape ecological interactions and challenge our understanding of ecosystem balance and resilience. Habitat fragmentation, pollution, altered resource availability, and selective species filtering often lead to novel community structures in these anthropogenically modified habitats (Shanahan et al. 2013). Birds have been extensively studied in human-dominated landscapes due to their high mobility and visibility, providing valuable insights into these ecological transformations.

In addition to their effects on biodiversity, human-modified habitats, including airport areas, may influence patterns of wildlife disease, potentially altering host–parasite dynamics and posing health risks. Changes in habitat structure and host community composition can affect disease transmission processes, with implications for zoonotic pathogen circulation among wildlife, domestic animals, and humans (Jones et al. 2008). Birds, in particular, play a significant role in the global transmission of infectious agents (Chang et al. 2020; Parums 2023), and parasites can substantially impact avian survival, development, and reproduction (Docherty and Long 1986; Bonier et al. 2007; Delgado-V. and French 2012; Giraudeau et al. 2014).

Understanding how parasites interact with avian hosts in disturbed habitats is critical for evaluating both conservation outcomes and public health risks. While many studies have explored the influence of urbanization on parasite abundance and host–parasite dynamics (Calegaro-Marques and Amato 2014; Jiménez-Peñuela et al. 2019; DeVore et al. 2020), fewer have examined these relationships specifically in the context of airports. Environmental factors associated with these habitats—such as reduced vegetation, altered microclimates, and pollution—can influence parasite prevalence and diversity (Chasar et al. 2009; Belo et al. 2012; Sehgal 2015; De Angeli Dutra et al. 2017; Fecchio et al. 2021a, b), particularly for haemosporidian parasites and other avian parasites whose persistence depends on their hosts, as their abundance is sensitive to ecological constraints experienced by avian hosts.

Haemosporidian parasites (Apicomplexa: Haemosporida) represent a widely distributed group of obligatory heteroxenous protozoans that parasitize amphibians, reptiles, birds, and mammals, relying on blood-feeding dipteran insects (Insecta: Diptera) as vectors. Avian malaria, caused by protozoa from the genus *Plasmodium* with mosquitoes (Diptera: Culicidae) serving as vectors, and haemoproteosis, caused by the genus *Haemoproteus* with biting midges and louse flies (Diptera: Ceratopogonidae and Hippoboscidae, respectively) as vectors, are among the main diseases attributed to these parasites, affecting a wide range of bird species (Valkiūnas 2005; Santiago-Alarcon et al. 2012).

Hematophagous vectors play a fundamental role in transmitting avian parasites, and their populations and behaviors are highly sensitive to environmental changes. In urban settings, some studies report reduced mosquito numbers, as observed in Spain (Ferraguti et al. 2016), while others reveal that certain mosquito species flourish in urbanized areas across the Americas, increasing transmission risks (Reyes et al. 2013; Aguilar et al., 2022; Duval et al. 2023). In addition, urbanization also influences ectoparasite dynamics in birds by altering nesting materials and introducing novel species interactions. For example, blue tits in highly developed areas have nests with fewer feathers and higher ectoparasite loads, suggesting that urban habitats impact parasite exposure (Reynolds et al. 2016). Likewise, invasive monk parakeets increase parasite loads in both their populations and cohabiting native pigeons, likely because crowding in urban areas fosters parasite-mediated competition and impacts local species health (Mori et al. 2019). Urban landscapes reshape disease dynamics by exposing vertebrate species to new vectors, particularly urban-adapted birds (Bradley and Altizer 2007), thereby amplifying transmission pathways and intensifying parasite pressures on urban bird populations, which alters ecological interactions.

A second key factor is avian host susceptibility, which can be markedly affected by anthropogenically modified environments such as those surrounding airports. Birds in these settings may experience diminished immune capacity, making them more susceptible to higher parasite loads (parasitemia) (Bradley and Altizer 2007; Costantini and Møller 2009). Airport environments expose birds to multiple physiological stressors—pollution, reduced vegetation cover, altered food availability, and especially chronic noise—which can collectively exacerbate disease severity (Giraudeau et al. 2014). For instance, birds inhabiting areas near airports are subject to intense and persistent noise levels, a major stressor known to disrupt communication, alter behavior, and impair physiological functioning (Alquezar et al., 2019). This constant noise pollution affects their ability to forage, reproduce, and avoid predators (Lowry et al. 2013). Airport noise is primarily composed of low-frequency sounds that overlap with and mask birds’ natural vocalizations. As a result, birds may expend additional energy to compensate for the acoustic interference, which can have adverse physiological consequences (Katti and Warren 2004).

Birds near airports have been observed altering their daily routines, such as advancing their morning choruses (Gil et al. 2015; Alquezar and Macedo 2019; Alquezar et al. 2020a). Disruption of the dawn chorus may reflect broader shifts in circadian rhythms, which have been linked to impaired immunocompetence (Cermakian et al. 2013). While such behavioral adaptations might buffer immediate stress impacts (McNamara and Buchanan 2005), chronic exposure to stressors can lead to physiological dysregulation and compromised homeostasis over time (Davis et al. 2008; Jenkins et al. 2013).

In birds experiencing chronic stress, the release of glucocorticoids, particularly corticosterone, is regulated by the hypothalamic–pituitary–adrenal (HPA) axis and typically follows a circadian rhythm to support metabolic functions and energy balance (Blas et al. 2005). Measuring corticosterone (CORT) levels in feathers provides a non-invasive means of assessing long-term HPA axis activity (Bortolotti et al. 2008). Although the use of feather CORT as a direct measure of chronic stress remains under discussion, it is widely accepted as an indicator of alterations in metabolic balance and energy regulation (Dickens and Romero 2013; Jimeno and Verhulst 2023).

This study compares haemosporidian parasite and ectoparasite prevalence in bird populations between airport-affected environments and quieter control areas in Brazil. In addition, we examine whether feather CORT levels and body condition are predictors of infection. Given the well-documented stressors associated with airport proximity, we posited that these birds would exhibit elevated haemosporidian parasite and ectoparasite prevalence in comparison to control areas, potentially stemming from compromised immune defenses. Although several disease-causing organisms often coexist within hosts, many studies have predominantly focused on individual infectious agents (Lello et al. 2018; Sweeny et al. 2021), providing a simplified picture of parasite–host dynamics. Here, we take a comprehensive approach because parasite coinfections have important implications for bird health and transmission of infections.

## Methods

### Study sites

**Fig. 1:**
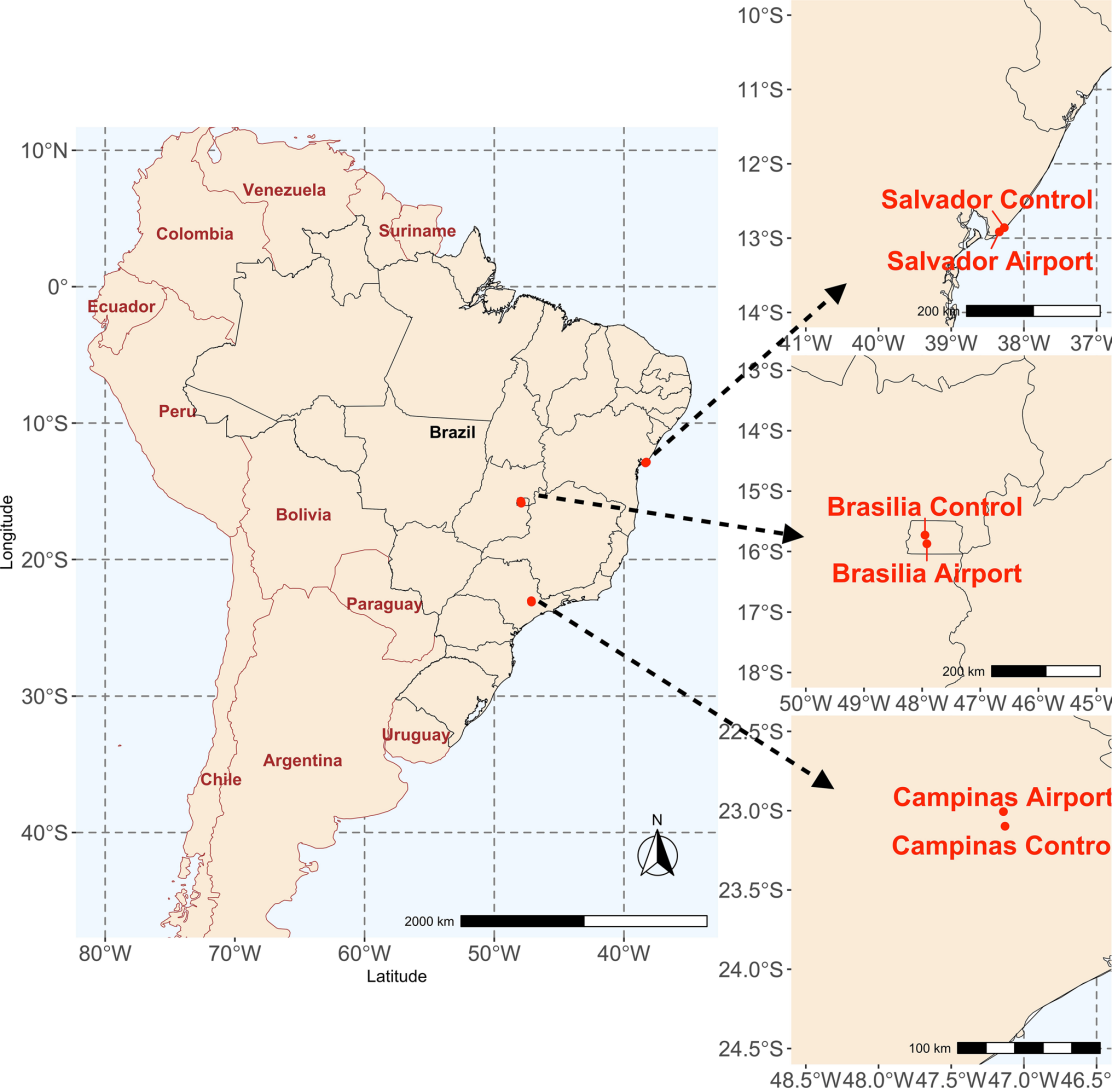
Locations where we sampled the individual birds: Brasília Airport: Presidente Juscelino Kubitschek International Airport; Brasília Control: Parque Nacional de Brasília; Compinas Airport: Viracopos International Airport; Campinas Control: Fazenda Santa Maria; Salvador Airport: Luís Eduardo Magalhães International Airport; Salvador Control: Condomínio Busca Vida

Three Brazilian airports were selected for the study based on their substantial aircraft activity and native vegetation surrounding their flight lanes. Specifically, we focused on forested areas affected by aircraft noise situated beyond the airport flight lanes. Each selected airport-affected region was paired with a corresponding silent control area, sharing comparable vegetation structures. These control areas were located 8 to 17 km from their respective airports. The airports under study were Presidente Juscelino Kubitschek International Airport in Brasília (− 15.872º, − 47.919º, Federal District), Viracopos International Airport in Campinas (− 23.006º, − 47.141º, São Paulo), and Luís Eduardo Magalhães International Airport in Salvador (− 12.916º, − 38.338º, Bahia). Corresponding silent control areas were: "Parque Nacional de Brasília" (− 15.728º, − 47.9511º), a private farm named "Fazenda Santa Maria" (− 23.098º, − 47.130º), and a residential area featuring significant protected green spaces, known as "Condomínio Busca Vida" (− 12.859º, − 38.270º) (Fig. 1). Viracopos Airport in Campinas handled about 10.5 million passengers in the first ten months of 2023. Salvador Airport recorded around 2 million passengers in the third quarter of 2024, and Brasília Airport served approximately 6.9 million passengers in the first half of 2024 (Fernandes et al. 2014; Baltazar et al. 2014; Rocha et al. 2016; de Oliveira et al. 2024). We selected these airports to encompass a geographically wide distribution across three distinct regions of Brazil, each characterized by different ecological features. Detailed descriptions of each study area, including habitat characteristics and disturbance levels, are available elsewhere (Alquezar et al. 2023). Despite regional ecological differences, all selected sites share key structural features: proximity to major transportation infrastructure, presence of adjacent green areas suitable for avian activity, and comparable climatic conditions during the sampling period, allowing for consistent cross-site comparisons of host–parasite dynamics. In addition, a previous study evaluated the bird species composition in all these locations. Species richness was higher in quiet-control sites than near airports. Among the common bird species, some adapted to airport conditions, showing greater abundance in these areas, while others avoided areas around airports, preferring quieter environments. Regional species pools influenced which species were present at airports. The prevalence of adaptable species in airport environments suggests an ongoing trend toward biotic homogenization, underscoring the often-overlooked impact of airports on bird communities and raising potential conservation concerns (Alquezar et al. 2020b).

The primary biome in these areas is Cerrado, which includes transitions with two other biomes. The "Cerrado sensu stricto" phytophysiognomy predominates within the Cerrado biome, marked by medium to tall trees and a moderate canopy cover (Ribeiro and Walter 1998). In the Campinas region, the Cerrado biome transitions to the Atlantic Forest biome, where taller trees and a denser canopy cover are prevalent. In Brasília, it is mostly “Cerrado senso stricto” biome. In contrast, the Salvador region exhibits a diverse blend of Cerrado, Atlantic Forest, and Caatinga biomes, characterized by medium to tall trees, moderate canopy cover, and sandy soil covered by shrubs and some xerophytic species (Almeida et al. 2015). In general, areas affected by airports exhibit a higher prevalence of urbanized landscapes in comparison to their quiet-control counterparts, which feature a more significant proportion of native landscapes (Alquezar and Macedo 2019). In Brasília, these sites encompass expansive land areas and exhibit the most extensive native landscapes. These regions stand out for their well-preserved Cerrado vegetation and forests near streams. On the other hand, sites within the other two areas, Salvador and Campinas, share similar total land extensions. Campinas Airport displays a landscape marred by the degradation of the Cerrado biome, characterized by modified areas primarily comprising grasslands and sparsely populated tree areas. In the control area in Campinas, the original Atlantic Forest vegetation has been affected by forest clearing to accommodate cattle and buffalo grazing, leading to the preservation of areas featuring grasslands and sparse trees for shade. The sites in Salvador represent a transition between the Cerrado biome and Restinga, a coastal ecosystem that extends along the Brazilian coast, associated with the Atlantic Forest biome (Castro et al. 2012). Restinga is interspersed with patches of forests and regions featuring dunes populated by smaller, less dense trees. Both sites have experienced degradation, manifesting as extensive clearings. The Salvador airport, in particular, is dominated by urbanized landscapes associated with airport facilities and the Brazilian Air Force.

### Data collection

We captured birds using ten 12-m mist nets during ten mornings at each site for five hours starting at dawn. We also occasionally captured birds using additional isolated nets and playback stimuli to increase sample sizes for a few species. In airport-affected areas in Brazil, mist nets were placed at a maximum distance of 250 m from flight lanes. Birds were captured during the peak of multiple breeding seasons: in the Brasília region (October 2014 and November 2015), the Campinas region (November–December 2014), and the Salvador region (December 2015–January 2016). We alternated sampling weekly between airport and control areas within each region.

Nets were regularly checked, and captured birds were placed in clean, breathable fabric bags so that feces could be collected in microtubes filled with ethanol 70% after birds were removed. We ringed birds with numbered metal rings (provided by CEMAVE-SNA/ICMBio) and obtained basic biometric information. Body mass was measured using a digital scale with 0.1-g precision, tarsus length was recorded with digital calipers accurate to 0.01 mm, and wing length was measured with a wing ruler to the nearest millimeter. Two or three tail feathers were collected for corticosterone analysis (Alquezar et al. 2023), and a blood sample was taken with sterile needles from the brachial vein and stored in microtubes filled with ethanol. Samples were frozen in the lab at -20ºC until analysis. All procedures were submitted and approved by the University’s Ethics Committee (129,022/2015 CEUA UnB), the Brazilian System of Authorization and Biodiversity Information (SISBIO 42578), the Brazilian Banding National System (CEMAVE-SNA 3856), and by the Brazilian National System for Management of Genetic Patrimony and Associated Traditional Knowledge (SISGEN AAA265A).

### Haemosporidian parasites

To screen for haemosporidian parasites, we transferred approximately 10 µL of blood samples into 1.5 mL microtubes, followed by dehydration at 37°C to prepare them for subsequent DNA extraction. We employed the conventional phenol–chloroform method for the extraction process, coupled with isopropanol precipitation (Sambrook and Russell 2001). The genomic DNA obtained was reconstituted in 50 µL of ultrapure water and quantified using a NanoDrop 2000 instrument (Thermo Scientific, Waltham, the United States). Between 50 and 100 ng of the extracted DNA was utilized in a screening PCR designed to amplify a 154-nucleotide segment of ribosomal RNA coding sequence within the mitochondrial DNA of *Plasmodium* and *Haemoproteus* in a single reaction. This PCR employed the primers 343F (5′-GCTCACGCATCGCTTCT-3′) and 496R (5′-GACCGGTCATTTTCTTTG-3′), following a protocol described elsewhere (Fallon et al. 2003).

A nested PCR was carried out for individuals yielding positive results; a nested-PCR was carried out to target the amplification of a 478 bp region within the cytochrome b gene. The first reaction involved primers HaemNFI (5′-AGACATGAAATATTATGGITAAG-3′) and HaemNR3 (5′-GAAATAAGATAAGAAATACCATTC-3′), with 50–100 ng of genomic DNA. A 1-μL aliquot of this PCR product from positive samples was subsequently employed as a template for the second reaction, using primers HaemF (5′-CTTATGGTGTCGA-TATATGCATG-3′) and HaemR2 (5′-CGCTTATCTGGAGATTGTAATGGTT-3′), following (Bensch et al. 2009). DNA from chickens' blood samples experimentally infected with *Plasmodium gallinaceum* served as positive controls, while ultrapure water was used as negative controls. The nested PCRs followed the protocol detailed in (Hellgren et al. 2004). Following successful nested PCR amplification, all positive products underwent purification using Polyethylene Glycol 8000 (Sambrook and Russell 2001) and were subjected to bi-directional sequencing with dye-terminator fluorescent labeling utilizing an ABI Prism 3100 sequencer (Applied Biosystems, Foster City, the United States). Sequencing chromatograms were scrutinized for the presence of mixed infections (signified by double peaks in the electropherograms) and aligned using ChromasPro software (Technelysium Pty Ltd, Helensvale, Australia). Assembled sequences were cross-referenced with data accessible in the MalAvi database (Bensch et al. 2009). For this study, sequences were regarded as distinct cytochrome b lineages when differing by one or more nucleotides.

### Ectoparasites

During biometric examinations, all captured birds underwent meticulous visual inspection to detect and remove ectoparasites. Whenever present, these ectoparasites were carefully extracted and preserved in microtubes containing 70% ethanol. The specimens were then forwarded to specialists for identification, with the aim of classifying them to the lowest possible taxonomic level. The ectoparasites examined included ticks (*Amblyomma* spp., Metastigmata: Ixodidae), chigger mites (Trombiculidae), hippoboscid flies (Diptera: Hippoboscidae), and botfly larvae (Diptera: Oestridae) (Arzua et al. 2005; Magalhães-Matos et al. 2017). Neither Mallophaga nor feather mites were collected. Tick nymphs of *Amblyomma* were identified under a stereomicroscope based on morphological criteria (Martins et al. 2010), whereas larvae could not be identified due to the lack of available dichotomous keys. Chigger mite specimens belonged to the genera *Blankaartia* sp. and *Eutrombicula* sp. (Bassini-Silva et al. 2023).

### Feather corticosterone

Two tail feathers were plucked and placed in uniquely labeled paper bags for each individually marked bird. These feathers were subsequently subjected to an extraction process to assess the long-term deposition levels of corticosterone (CORT) following Alquezar et al. (2023). The extraction procedure followed a modification of a methanol-based extraction (Bortolotti et al. 2008). Extracts were assayed with a CORT-specific EIA (enzyme immunoassay) procedure (DRG Instruments GmbH, Marburg, Germany), and plates were read with a spectrophotometer (Biotek Instruments Inc, Winooski, VT, USA). Final concentrations were expressed as CORT picograms by feather length (mm). See Alquezar et al. (2023) for more details on the procedure.

### Body condition and bird phylogeny

Body condition was estimated by using the residuals derived from a mass-body length regression model (McGraw et al. 2020). Given the potentially influential spatial variability in body condition (de Souza Penha and da Silva Rodrigues 2022; Aguiar de Souza Penha and De La Torre 2024), we conducted regression analyses on a per-species and per-location basis. We restricted analyses to species with a minimum of five captures to increase model reliability. We used the Birdtree (Jetz et al. 2012) as our bird phylogeny in the statistical models (see more in the Statistical Analysis section) and kept bird species identity as in Birdtree.

### Statistical analysis

To investigate whether proximity to airports influences the occurrence of haemosporidian parasites and ectoparasites in wild birds, we fit a series of Bayesian phylogenetic multilevel models using the brm function from the brms package (Brooks et al. 2017; Bürkner 2017, 2018, 2021). We modeled parasite presence (1) or absence (0) as the response variable using a Bernoulli distribution. Separate models were constructed for each parasite group (haemosporidians and ectoparasites).

Fixed effects included area type (airport-affected vs. quiet-control sites), city (Salvador, Campinas, and Brasília), and their interaction. The interaction term was retained in the final models only when supported by the posterior distribution. To account for phylogenetic non-independence among species, we included a species-level random effect using a phylogenetic covariance matrix A, derived from a pruned ultrametric tree with the vcv.phylo() function from the ape package. Species names in the dataset were matched to the tree’s tip labels, and all species included in the models were present in the phylogeny. We used default priors as returned by the get_prior() function in brms.

Each model was run with four chains and 10,000 iterations. Convergence was assessed using split R̂ values (with values ≈ 1 considered ideal) and effective sample sizes (> 1,000). To ensure model convergence, we examined binned plots relating the mean predictions and residuals for each model using the *binnedplot* function from the *arm* package (Gelman and Su 2022). Furthermore, we evaluated the split-Rhat and effective sample size estimates, considering values equal to one for split-Rhat and values exceeding 1,000 for effective sample size as indicators of well-fitted models (Gelman et al. 2013). We assessed model performance using the root mean square error (RMSE), with values closer to 0 indicating better predictive accuracy, and evaluated calibration visually using a binned residual plot.

We then applied the same modeling framework to a subset of 476 individuals from 13 species for which we had feather corticosterone (CORT) and body condition data. This subset was determined by funding limitations, which constrained the number of samples that could be processed for both physiological and parasitological analyses. In these models, we tested whether parasite occurrence could be predicted by log-transformed feather CORT concentrations, normalized body condition (derived from species- and site-specific residuals of mass ~ tarsus length regressions), and the presence of the other parasite group (i.e., haemosporidian presence included as a predictor in the ectoparasite model, and vice versa).

These models also included interactions between treatment and both CORT and body condition to assess whether physiological effects varied by site type. City and treatment were retained as main effects, and the same phylogenetic random effect structure was applied.

Posterior distributions were analyzed, and predictors were considered meaningful when the posterior probability of direction exceeded 95% and the 95% credible interval did not overlap zero. All analyses were conducted in R (R Core Team 2019).

## Results

### Haemosporidian parasites

We captured a total of 1,096 individual birds, representing a diverse range of 100 bird species, spread across 27 different families and spanning nine distinct orders (Supplemental Table 1). We observed an overall prevalence of haemosporidian parasite infection of 21.8% (240 infected individuals within 1,096 total individuals) from all locations (Fig. 1). These infections were found in 49 different bird species, representing 49% of the total 100 species surveyed. The occurrences varied widely among the species; *Turdus leucomelas* had the highest prevalence of haemosporidian parasite infection (44%; *n* = 70 individuals). Additionally, we identified several bird species with only a single individual being infected with haemosporidian parasites (Supplemental Tables 2; 3). Further analysis revealed that out of the 240 infected individuals, 63% were infected with *Plasmodium* spp. (41 of 65 unique parasite lineages), 34% with *Haemoproteus* (*Parahaemoproteus*) (22 of 65 total parasite lineages), and only 3% with *Haemoproteus* (*Haemoproteus*) parasite lineages (2 of 65 total parasite lineages). Summary results of haemosporidian parasite lineages can be found in Supplementary Table 4.

**Table 1:** Bayesian multilevel models with ectoparasite, haemosporidian parasite occurrence (*Plasmodium* and *Haemoproteus Parahaemoproteus* combined), *Plasmodium* occurrence only, and the *Haemoproteus Parahaemoproteus* occurrence only as response variables. Treatment (area type) and city are used as predictors

Variable	Estimate	SE	lower 95%	upper 95%	Rhat	ESS
Model: Haemosporidian parasite occurrence ~ Treatment + City						
InterceptA	0.37	0.13	**0.13**	**0.65**	1.00	1181
Treatment (control)	0.22	0.09	**0.04**	**0.40**	1.00	2824
City (Campinas)	0.26	0.12	**0.03**	**0.49**	1.00	2750
City (Salvador)	− 0.23	0.11	**− 0.45**	**− 0.01**	1.00	2388
Random term (bird phylogeny)	0.02	0.01	**0.00**	**0.04**	1.00	1728
Model: Ectoparasite occurrence ~ Treatment * City						
InterceptA	− 2.61	0.43	**− 3.54**	**− 1.78**	1.00	1191
Treatment (control)	1.55	0.30	**0.98**	**2.17**	1.00	2716
City (Campinas)	**− **0.62	0.49	**− **1.64	0.30	1.00	2730
City (Salvador)	**− **1.62	0.70	**− 3.05**	**− 0.40**	1.00	2065
Treatment (control) * City (Campinas)	**− **1.40	0.65	**− 2.66**	**− 0.15**	1.00	3220
Treatment (control) * City (Salvador)	**− **1.04	0.86	**− **2.69	0.65	1.00	2625
Random term (bird phylogeny)	0.06	0.04	**0.01**	**0.16**	1.00	1231

^A^Reference values are airport for the treatment variable and Brasilia for the city variable

Estimate, standard error (SE), the lower 95% confidence interval (l–95%), the upper 95% confidence interval (u-95%), the Rhat, and the effective sample size estimates (ESS) are displayed. Statistically significant variables are marked in bold. Asterisk means the interactive term between treatment and city (which was not significant for the haemosporidian parasite occurrence model)

### Ectoparasites

We observed ectoparasite infestations in 101 individuals, corresponding to an overall prevalence of 9.2%. Among bird species, *Elaenia chiriquensis* (111 examined) showed the highest number of infested individuals, with 17 cases in the control area and 2 in the airport area (Supplementary Table 3). Trombiculid mites (*Blankaartia* sp. and *Eutrombicula* sp.) were the most prevalent ectoparasites, occurring in 58 individuals, followed by immature ticks of the genus Amblyomma (36 nymphs and 11 larvae), which infested 32 individuals. Hippoboscid flies were comparatively rare, recorded in only three individuals. In some cases, birds carried multiple ectoparasite families simultaneously. For example, one *Synallaxis frontalis* from the control area in Brasília was parasitized by both *Amblyomma* (Ixodidae) and Hippoboscidae, while seven individuals of seven different species, also from the control area in Brasília, carried both Ixodidae (*Amblyomma*) and Trombiculidae (Supplementary Table 5).

### Co-occurrence of haemosporidian parasites and ectoparasites

Our data show that 15 individuals were found to harbor both haemosporidian parasites and ectoparasites (Supplementary Table 6). *Turdus leucomelas* stood out as the species with the highest number of such instances. Notably, these co-occurrences were primarily observed in individuals captured within the control areas of Salvador and Campinas.

### Parasitism and site type

**Fig. 2:**
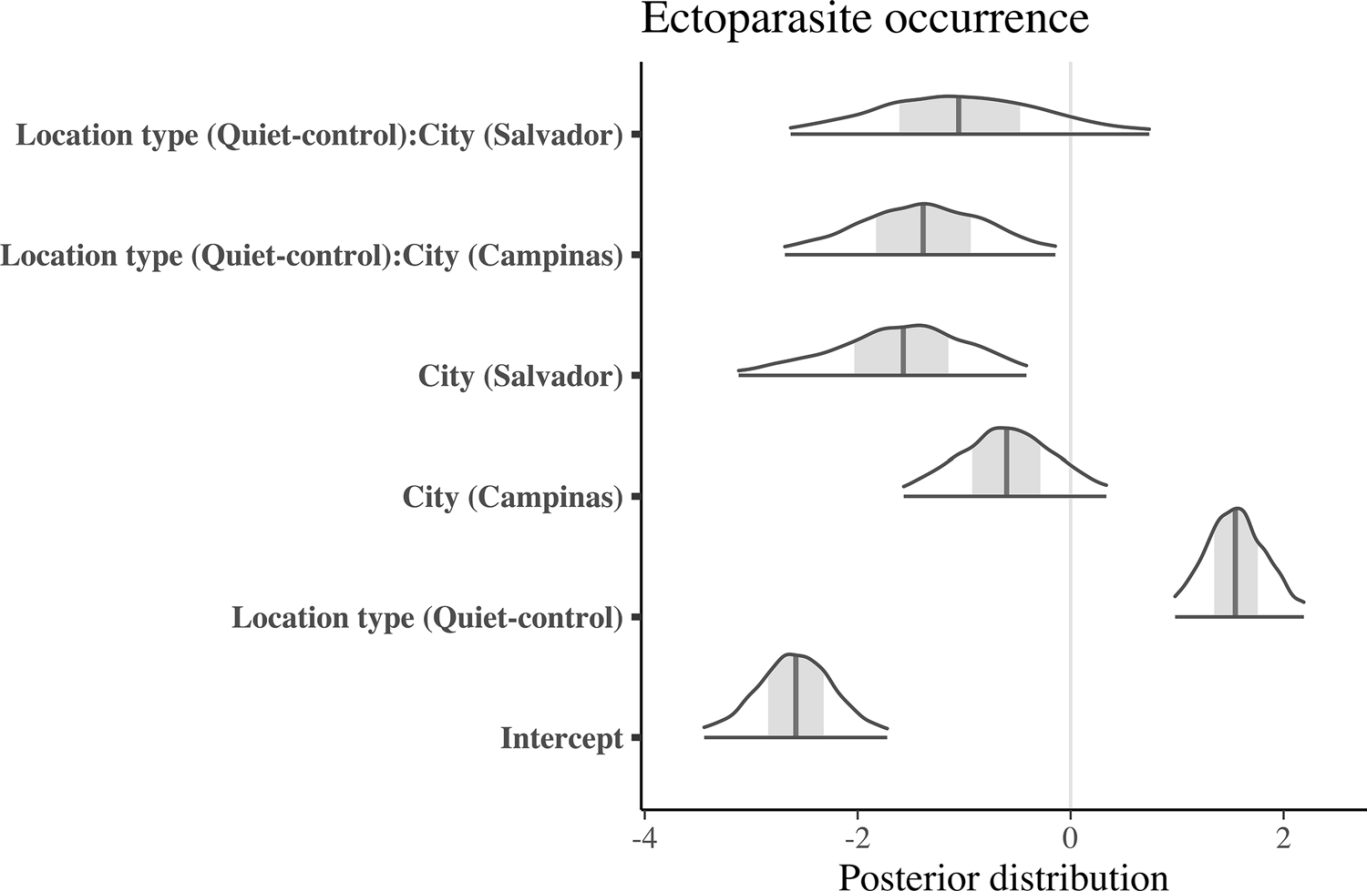
Posterior distribution from the Bayesian multilevel model with the ectoparasite occurrence as the response variable

**Fig. 3:**
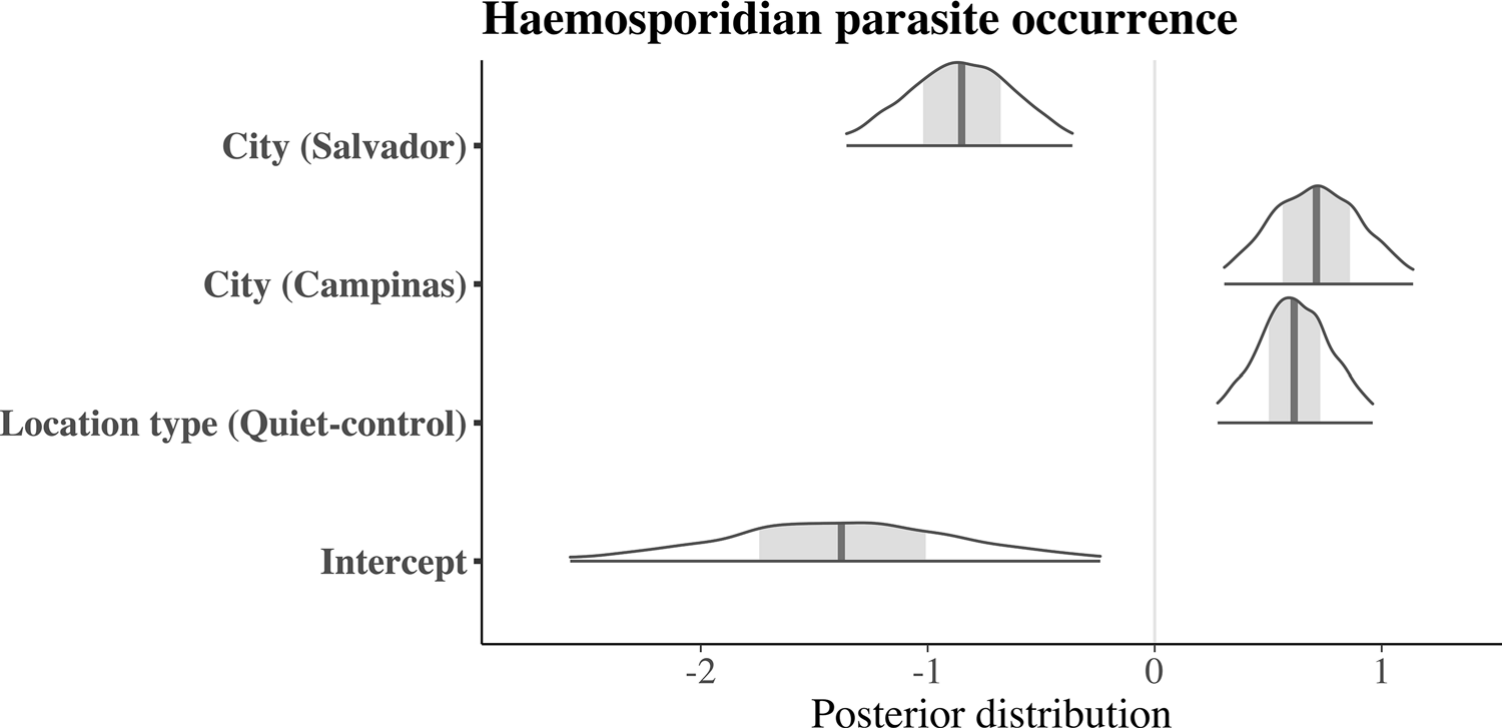
Posterior distribution from the Bayesian multilevel model with the haemosporidian parasite occurrence as the response variable

We found that the city was a significant predictor of haemosporidian parasite prevalence, with higher values in quiet-control than in airport-affected sites across all cities (Table 1; Figs. 2 and 3). More specifically, there was an elevated probability of haemosporidian parasite occurrence in Campinas and Brasilia, with Brasilia recording the highest prevalence of infected individuals. In contrast, Salvador exhibited the lowest occurrence of haemosporidian parasites, irrespective of the area type.

**Fig. 4:**
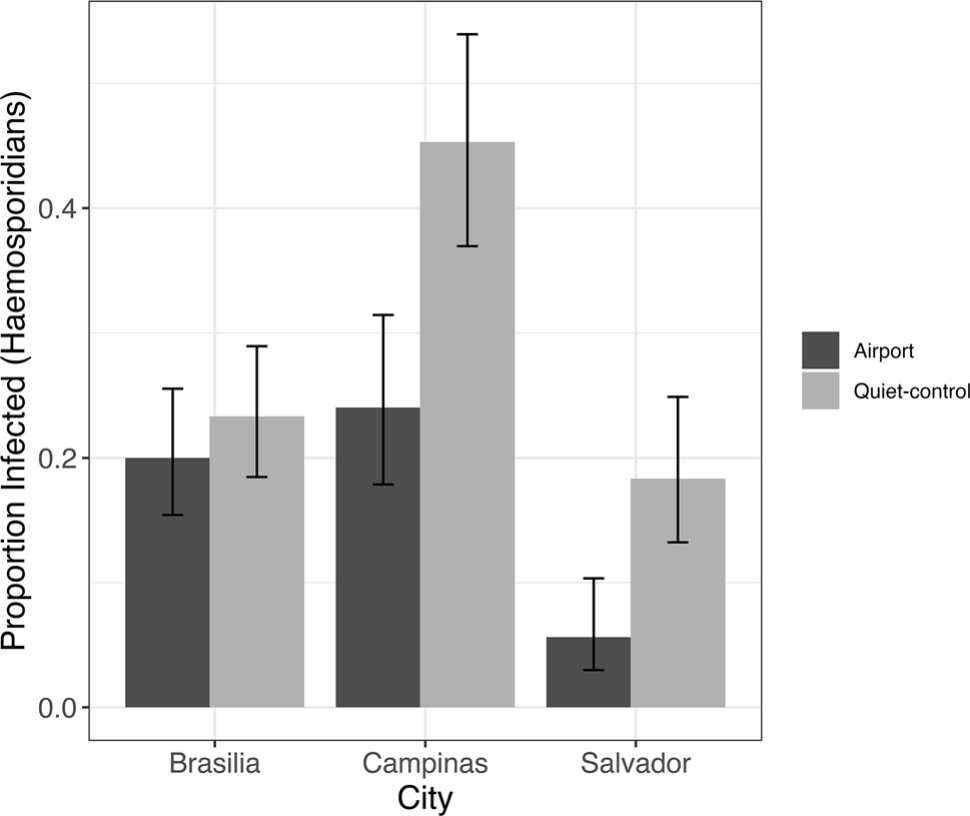
Proportion of individuals infected with haemosporidian parasites by treatment (airport vs. quiet-control) and city (Brasília, Campinas, and Salvador). Bars represent prevalence estimates with 95% confidence intervals

**Fig. 5:**
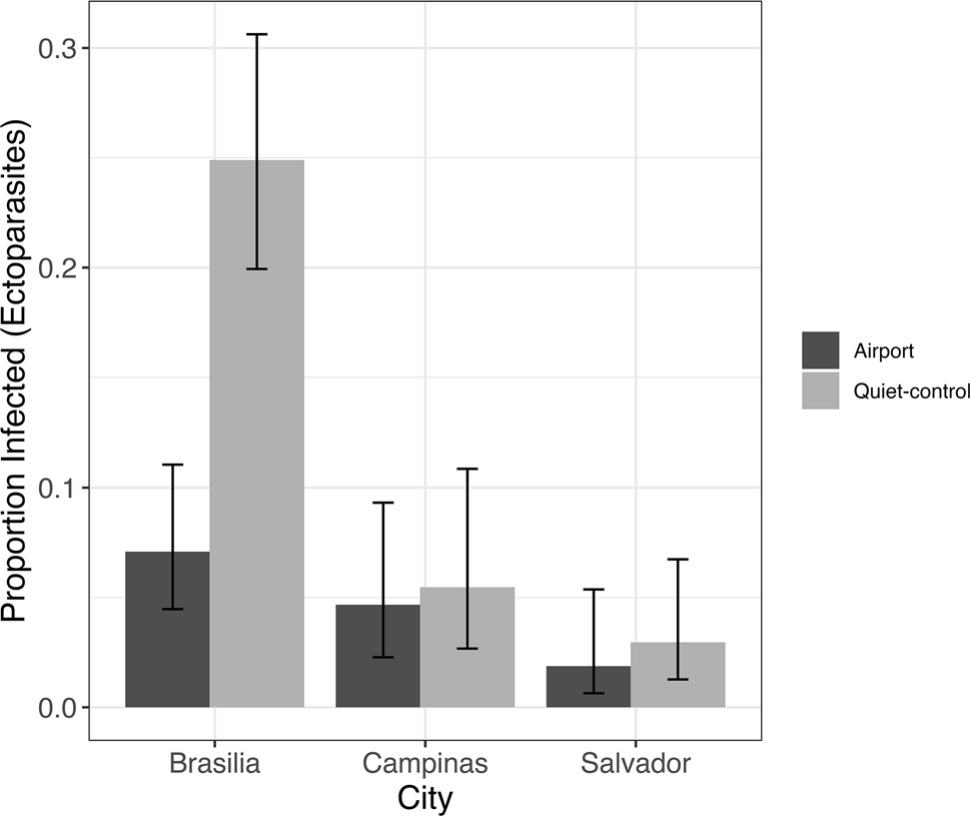
Proportion of individuals infested with ectoparasites by city and treatment type (grouped bars: airport vs. quiet-control). Bars represent prevalence estimates with 95% confidence intervals

Regarding ectoparasites, our analysis revealed a more complex pattern with a significant interaction between area type and city. Individuals at the quiet-control site in Brasilia exhibited a higher likelihood of carrying ectoparasites than those in the airport-affected area (Figs. 4, 5; Table 1). Conversely, in Campinas and Salvador, there appeared to be minimal disparity in infection levels between quiet-control and airport-affected locations (Figs. 4, 5; Table 1). We evaluated model performance using RMSE, which was 0.277 for the ectoparasite model and 0.408 for the haemosporidian parasite model, indicating low error rates and good predictive accuracy for both models.

### Corticosterone, body condition, and parasitism

**Fig. 6:**
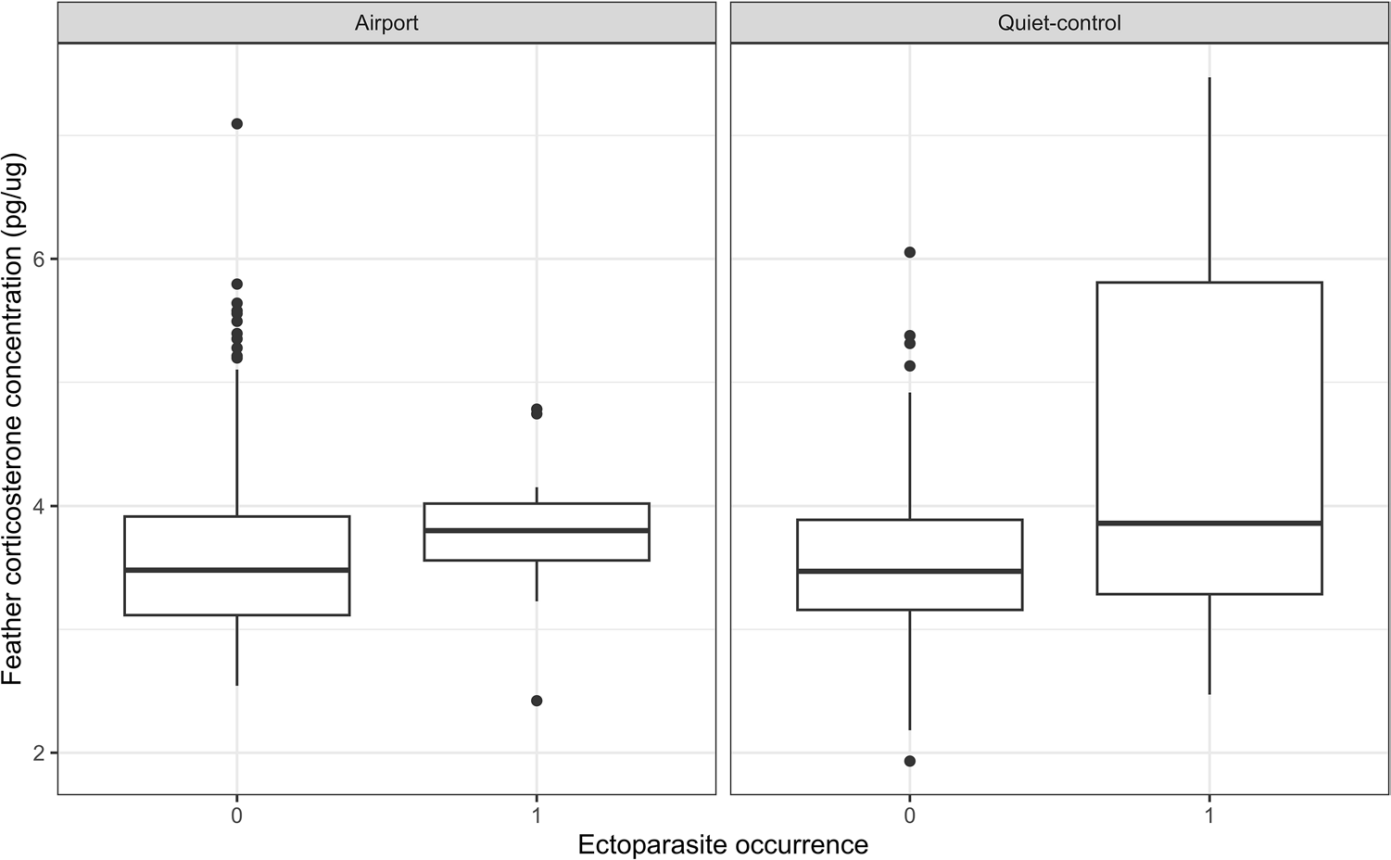
Relationships between ectoparasite occurrence and CORT in quiet-control areas (right) and in airports (left). Different letters indicate statistically significant differences. Numbers indicate ectoparasite absence ("0") and presence ("1")

We found an association among location type, CORT, and ectoparasite occurrence (Table 2). Individuals from quiet-control areas carrying ectoparasites exhibited a higher corticosterone concentration (Fig. 6). However, we found no compelling evidence to suggest that CORT, body condition, treatment, or the co-occurrence of ectoparasites was associated with the presence of haemosporidian parasites (Table 2). We evaluated model performance using RMSE, which was 0.283 for the ectoparasite model and 0.417 for the haemosporidian parasite model, indicating that the ectoparasite model had greater predictive accuracy.

**Table 2:** Bayesian multilevel models showing two models with the haemosporidian parasite and ectoparasite occurrence as the response variables. Corticosterone concentration in feathers, body condition, parasite coinfection were used as predictors, as well as the interaction between location type and numeric variables

Variable	Estimate	SE	lower 95%	upper 95%	Rhat	ESS
Model: Haemosporidian parasite occurrence ~ CORT * location type + body condition * location type + ectoparasite occurrence						
InterceptA	− 2.99	1.49	**− 6.05**	**− 0.06**	1.00	2019
Corticosterone concentration	0.28	0.30	− 0.32	0.86	1.00	22.53
Area type (quiet -control areas)	2.02	1.28	− 0.51	4.55	1.00	2450
Body condition	− 0.11	0.16	− 0.43	0.21	1.00	2674
Haemosporidian parasite occurrence (presence)	− 0.64	0.51	− 1.68	0.31	1.00	2966
Corticosterone concentration * area type (quiet -control areas)	− 0.28	0.35	− 0.97	0.41	1.00	2221
Body condition * area type (quiet-control areas)	0.10	0.25	− 0.39	0.58	1.00	2598
Random term (bird phylogeny)	0.26	0.09	**0.13**	**0.46**	1.00	1690
Model: Ectoparasite occurrence ~ CORT * location type + body condition * location type + haemosporidian parasite occurrence						
InterceptA	− 4.59	1.75	− **8.07**	− **1.22**	1.00	2639
Corticosterone concentration	0.42	0.41	-0.39	1.21	1.00	2753
Area type (quiet -control areas)	− 2.42	1.88	− 6.11	1.07	1.00	2007
Body condition	− 0.16	0.24	− 0.64	0.31	1.00	2750
Haemosporidian parasite occurrence (presence)	− -0.68	0.49	− 1.66	0.25	1.00	2704
Corticosterone concentration * area type (quiet -control areas)	0.92	0.49	**0.02**	**1.90**	1.00	2021
Body condition * area type (quiet-control areas)	0.05	0.38	-0.69	0.80	1.00	2823
Random term (bird phylogeny)	0.18	0.09	**0.05**	**0.40**	1.00	1157

Variable, estimate, standard error (SE), the lower 95% confidence interval (l–95%), the upper 95% interval (u-95%), the Rhat, and the and the effective sample size estimates (ESS) are displayed. Statistically significant variables are marked in bold

^A^Reference level of haemosporidian parasite occurrence (absent); and ectoparasite occurrence (absent)

## Discussion

Our study uncovered significant ecological patterns of interactions between bird species and parasite infections in airport versus less-disturbed control areas. Notably, some birds harbored both haemosporidian parasites and ectoparasites, with prevalence varying significantly by city and area type. Contrary to our predictions, haemosporidian parasite infections were more common in quiet-control areas across all cities, and quiet-control locations in Brasilia had a higher prevalence of ectoparasites. Our analysis also indicated that CORT was a significant predictor of ectoparasite occurrence in quiet-control areas, with individuals harboring ectoparasites exhibiting higher CORT levels, a pattern not observed in airport-affected locations.

Contrary to our predictions, haemosporidian prevalence was higher in quiet-control areas across all cities, with Brasília showing the highest proportion of infected individuals and Salvador the lowest. This pattern challenges the stress–immunocompromise hypothesis, which predicts higher infection risk in disturbed environments (Alquezar et al. 2020a). Recent meta-analyses have similarly found that urban birds are not systematically more challenged in terms of health, hormone levels, or immune status than their rural counterparts (2024),Iglesias-Carrasco et al. 2020; Reid et al. 2024 suggesting that populations persisting in human-modified habitats may already be adapted to such conditions. Similar patterns have been reported for *Volatinia jacarina*, with haemosporidian prevalence being greater in habitats with native vegetation cover than in more urbanized areas (Fecchio et al. 2021c). Our results therefore suggest that airports may provide less optimal conditions for haemosporidian transmission. Three non-mutually exclusive mechanisms could explain this: (a) vector control protocols in dengue-endemic regions may reduce conditions favorable for parasite vectors (Fares et al. 2015); (b) airports may offer fewer suitable microhabitats for vector proliferation (Lowry et al. 2013); and (c) bird populations persisting in these environments may be better adapted to infection, limiting parasite establishment (Delgado-V. and French 2012). While plausible, these explanations remain speculative due to the absence of vector abundance or control data. Future research should integrate vector surveillance and management records to directly assess these mechanisms.

The high ectoparasite infestation observed only in the Brasília quiet-control area highlights the ecological role of wild birds in carrying ectoparasites that can serve as vectors of zoonotic diseases (Luz et al. 2017). This site’s Cerrado and transitional vegetation may provide more favorable habitats for ectoparasites than the more urbanized or forested environments of Campinas and Salvador (Laporta et al. 2013). In the Neotropics, birds are important hosts for immature stages of *Amblyomma* ticks, which complete their life cycle on mammals such as *Coendou* sp. (*Amblyomma longirostre*) and *Tamandua tetradactyla* (*A. nodosum*) (Szabó et al. 2013; Luz et al. 2017). By harboring immature stages, birds help sustain *Amblyomma* populations, some of which are known vectors of spotted fever group rickettsiae (Torga et al. 2013; Ogrzewalska and Pinter 2016; Bassini-Silva et al. 2018). Therefore, the particularly high prevalence of ectoparasites in the control area of Brasília may reflect both local habitat structure and vector ecology. The Cerrado biome and its transitional vegetation could offer more favorable ecological conditions for ectoparasites compared to the more urban or forested environments of the other cities. These findings underscore the complex interplay between landscape type and ectoparasite occurrence, suggesting that highly urbanized environments such as airports may be less conducive to the proliferation of certain parasite groups, depending on local environmental conditions.

We also found that feather corticosterone (CORT) predicted the occurrence of ectoparasites in quiet-control locations but not in airport-affected sites. Specifically, individuals trapped in quiet-control areas and infested with ectoparasites exhibited higher CORT levels. This pattern is consistent with findings in *Tachycineta bicolor* and *Aquila chrysaetos*, which showed elevated CORT levels in parasitized individuals (Harriman et al. 2014; Dudek et al. 2021), suggesting a link between glucocorticoid levels and ectoparasite-related stress. The absence of this relationship in airport birds may reflect a reduced physiological responsiveness to ectoparasites in chronically disturbed environments or a lower overall parasite burden insufficient to trigger a stress response. Alternatively, it could suggest that ectoparasites act as a more potent or detectable stressor in relatively natural habitats, where baseline CORT is lower and variation is more informative. One possibility is that birds in airport environments are exposed to chronic low-level stressors (e.g., noise, disturbance) that dampen their hormonal responsiveness to parasites. Alternatively, individuals in airport sites may differ behaviorally or physiologically, perhaps through habituation or selection for stress-tolerant phenotypes, resulting in weaker CORT responses when ectoparasites are present. Notably, the consistent absence of a CORT–ectoparasite relationship across all airport sites may reflect underlying similarities in disturbance regimes, habitat structure, or management practices common to major transportation hubs, which could homogenize physiological responses despite regional ecological differences. Passenger volume alone may not fully capture the complexity of environmental disturbance, as factors such as noise intensity, habitat fragmentation, and human activity patterns likely interact in shaping host physiology and parasite exposure. This pattern aligns with our broader finding of lower overall CORT and reduced parasite prevalence in airport sites, although the magnitude of this effect varied among cities, suggesting that environmental context shapes both exposure risk and physiological responses. Overall, the CORT–ectoparasite relationship appears to be complex and species- or context-dependent.

Previous work on Sturnus vulgaris, for instance, has shown both negative (Pryor and Casto 2017) and null (Pryor and Casto 2015) associations between ectoparasite burden and glucocorticoids. Such variation may reflect differences in host resistance, tolerance, or compensatory mechanisms (St. Juliana et al. 2014). In contrast, haemosporidian parasite occurrence was not associated with variation in feather CORT concentrations. This is consistent with previous work on *Lepidothrix coronata*, which also found no significant association between haemosporidian infection and CORT levels (Bosholn et al. 2019). We also found no evidence that proximity to airports influenced baseline stress levels, as measured by either feather CORT or body condition. Infected individuals living near airports did not show higher CORT concentrations than those in control areas. While feather CORT is a valuable integrated measure of glucocorticoid output over time, its precision as a stress indicator remains debated (Dickens and Romero 2013; Jimeno and Verhulst 2023). Our interpretation considers this limitation and highlights the need for future studies to complement CORT with additional physiological or behavioral indicators. The most surprising result was the lower prevalence of haemosporidian parasites and ectoparasites in airport sites, which runs counter to the predictions of the stress–immunocompromise hypothesis. This counterintuitive pattern suggests that urban pressures do not necessarily lead to higher parasite risk. Instead, several mechanisms may contribute to reduced infection at airports. For example, vector control measures in dengue-endemic areas may inadvertently suppress avian parasite transmission. Additionally, airports may provide fewer microhabitats suitable for vector development (Lowry et al. 2013), or the structure and materials of airport landscapes may be less conducive to vector survival. It is also possible that the bird species inhabiting these environments are more stress-tolerant or parasite-resistant, which could reduce both prevalence and intensity of infection.

Our study relied on parasite prevalence data (presence/absence), and it is possible that incorporating infection intensity could have altered some of our conclusions. Intensity measures, such as mean parasite load per infected host, provide critical insights into the physiological costs of infection and the magnitude of transmission potential across environments (Bush et al. 1997; Margolis et al. 1982). Nevertheless, molecular diagnostics such as PCR are highly sensitive and specific, even at low intensities, making prevalence a reliable proxy for ecological inference. Previous studies show that prevalence data can track ecological and temporal patterns that are also revealed by intensity (Han et al. 2023; Podmokła et al. 2014) although other work demonstrates that intensity data can uncover distinct or complementary trends (Reinoso-Pérez et al. 2016). To refine these inferences, future studies should quantify parasitaemia or parasite load, ideally using quantitative PCR (qPCR), to assess whether observed prevalence differences reflect reduced infection pressure or simply lower infection intensities that evade detection (Marani et al. 2021; Seidl et al. 2024). Pairing prevalence with intensity data, as well as measures such as ectoparasite burden and vector surveillance, would allow clearer separation of ecological processes driving variation in infection risk from the physiological costs borne by hosts.

Our findings are consistent with emerging literature showing that urbanization can sometimes reduce, rather than increase, parasite exposure (Fecchio et al. 2021a). The relevance of our results is constrained by the correlational nature of the study and the absence of direct mechanistic data. Longitudinal or experimental designs that integrate host physiology, vector ecology, and urban landscape features will be critical for disentangling the drivers of parasite dynamics in modified environments.

Our study revealed a 21.8% prevalence of haemosporidian parasites among all screened birds, consistent with other studies in Brazil (Ribeiro et al. 2020; Rodrigues et al. 2021), despite some reporting higher (Belo et al. 2011; Aguiar de Souza Penha et al. 2023; de Angeli Dutra et al. 2023) or lower prevalence (Fecchio et al. 2013; Anjos et al. 2023). This highlights the variability in haemosporidian parasite and avian host interactions across different locations. Additionally, we found a low overall prevalence of ectoparasite infestation, consistent with previous studies (Cantarin Neiva et al. 2021). The Trombiculidae family was the most frequent ectoparasite, aligning with prior findings (Silva 2017; Bassini-Silva et al. 2018; but see Dantas-Torres et al. 2021). Notably, *Elaenia chiriquensis*, a migratory species captured in Brasília, had the highest number of ectoparasites among the birds sampled. This finding suggests a potential role in the life cycle of ticks and Trombiculidae mites, with its migratory behavior potentially amplifying the impact on parasite spread (de Angeli Dutra et al. 2021). We also identified some co-occurrence of haemosporidian parasites and ectoparasites in quiet-control areas in Salvador and Campinas. While a previous study did not find a relationship between these parasites (de Angeli Dutra et al. 2023), our results suggest that co-occurrence may happen, particularly in *Turdus leucomelas*. Therefore, we recommend future studies focused on *Elaenia chiriquensis* for its high ectoparasite prevalence as well as its migratory behavior and *Turdus leucomelas* for its notable co-occurrence of haemosporidian parasites and ectoparasites. This could enhance our understanding of their roles as disease reservoirs and the effects of parasitism on their physiological and immune parameters. One limitation of our study is the absence of infection intensity data (e.g., parasitemia levels), which, while complementary to prevalence, would have provided deeper insights into the physiological burden of infection and potential fitness costs for hosts.

In summary, our study revealed significant ecological interactions between bird species and parasite infections across airport-affected and quiet-control areas, highlighting complex spatial dynamics and species-specific responses. Some birds harbored both haemosporidian parasites and ectoparasites, with prevalence varying by city and area type. Ectoparasite prevalence was highest in the quiet-control site of Brasília, while haemosporidian infections were more common in quiet-control areas across all cities. Additionally, CORT levels were positively correlated with ectoparasite occurrence, but only in quiet-control areas, suggesting context-dependent physiological responses. Our results raise the possibility that well-managed airports may serve as partial barriers to parasite transmission—underscoring their potential role in integrated public health and wildlife surveillance strategies. Because this was an observational study, we cannot determine whether elevated CORT is a physiological response to parasitism or a predisposing condition that increases susceptibility. We recommend that future research address this question using experimental approaches to clarify causality. Overall, our findings suggest that urbanized environments and airports may harbor fewer avian parasites, possibly due to effective vector control protocols or less favorable conditions for vector development. This challenges the assumption that birds in noisy, human-modified environments are necessarily more stressed or immunocompromised.

## Author contribution statement

The idea was originally formulated by GTP, RHM, and DG. The methodology was developed and implemented by GTP, RDA, FCF, DdAD, and EMB. Data analysis, result interpretation, and the first draft were carried out by VASP, GTP, and DG. All authors contributed to analyzing and reviewing the final drafts.

## Supplementary Information

Below is the link to the electronic supplementary material.

Supplementary file1 (DOCX 74 KB)

## Data Availability

The datasets used and/or analyzed during the current study are available from the corresponding author on reasonable request.

## Code availability

R codes used during the current study are available from the corresponding author on reasonable request.
