# Supplementary material for: Ectoparasite and blood parasite prevalence in birds: a comparative study between urbanized airport environments and natural habitats
Source: Oecologia. 2025 Oct 11;207(11):172. doi: 10.1007/s00442-025-05811-3 (PMC12515230; doi:10.1007/s00442-025-05811-3)
Supplement: Supplementary file 1 — Supplementary file1 (DOCX 74 KB) [file 442_2025_5811_MOESM1_ESM.docx]

Supplemental material

Supplementary Table 1: Order, family, species identity, and the quantity of captured individuals in each location (SA: Salvador’s airport; SC: Salvador’s control area; CA: Campinas’ airport; CC: Campinas’ control area; BA: Brasilia’s airport; and BC: Brasilia’s control area).

| Order | Family | Species | SA | SC | CA | CC | BA | BC |
| --- | --- | --- | --- | --- | --- | --- | --- | --- |
| Accipitriforme | Accipitridae | *Buteo magnirostris* | 0 | 0 | 0 | 0 | 1 | 0 |
| Caprimulgiformes | Caprimulgidae | *Caprimulgus maculicaudus* | 0 | 0 | 0 | 0 | 2 | 1 |
|  |  | *Caprimulgus parvulus* | 0 | 0 | 0 | 1 | 1 | 0 |
|  |  | *Nyctidromus albicollis* | 0 | 2 | 0 | 0 | 0 | 0 |
| Columbiformes | Columbidae | *Columbina minuta* | 1 | 0 | 0 | 0 | 0 | 0 |
|  |  | *Columbina passerina* | 0 | 3 | 0 | 0 | 0 | 0 |
|  |  | *Columbina squammata* | 0 | 1 | 0 | 0 | 0 | 0 |
|  |  | *Columbina talpacoti* | 3 | 2 | 2 | 1 | 1 | 0 |
|  |  | *Leptotila rufaxilla* | 0 | 0 | 0 | 1 | 0 | 0 |
|  |  | *Leptotila verreauxi* | 0 | 0 | 1 | 1 | 0 | 0 |
| Cuculiformes | Cuculidae | *Crotophaga ani* | 6 | 0 | 0 | 0 | 5 | 0 |
| Galbuliformes | Galbulidae | *Galbula ruficauda* | 0 | 0 | 0 | 0 | 1 | 0 |
|  | Bucconidae | *Nystalus maculatus* | 0 | 2 | 0 | 0 | 0 | 0 |
| Passeriformes | Dendrocolaptidae | *Lepidocolaptes angustirostris* | 0 | 0 | 0 | 0 | 2 | 6 |
|  | Fringilidae | *Euphonia chlorotica* | 0 | 0 | 0 | 0 | 2 | 0 |
|  | Furnariidae | *Furnarius rufus* | 1 | 1 | 0 | 0 | 2 | 1 |
|  |  | *Pseudoseisura cristata* | 0 | 1 | 0 | 0 | 0 | 0 |
|  |  | *Synallaxis albescens* | 0 | 0 | 0 | 0 | 0 | 6 |
|  |  | *Synallaxis frontalis* | 0 | 0 | 14 | 4 | 2 | 2 |
|  |  | *Synallaxis spixi* | 0 | 0 | 1 | 1 | 0 | 0 |
|  | Hirundinidae | *Stelgidopteryx ruficollis* | 0 | 0 | 0 | 2 | 0 | 0 |
|  | Icteridae | *Icterus cayanensis* | 0 | 1 | 0 | 0 | 0 | 0 |
|  | Mimidae | *Mimus gilvus* | 4 | 3 | 0 | 0 | 0 | 0 |
|  |  | *Mimus saturninus* | 0 | 0 | 0 | 0 | 1 | 0 |
|  | Parulidae | *Basileuterus culicivorus* | 0 | 0 | 0 | 1 | 0 | 0 |
|  |  | *Basileuterus leucophrys* | 0 | 0 | 0 | 0 | 1 | 0 |
|  |  | *Geothlypis velata* | 0 | 0 | 0 | 0 | 1 | 0 |
|  | Passerellidae | *Ammodramus humeralis* | 0 | 0 | 1 | 2 | 3 | 3 |
|  |  | *Arremon flavirostris* | 0 | 0 | 0 | 0 | 1 | 0 |
|  |  | *Zonotrichia capensis* | 0 | 0 | 7 | 16 | 1 | 25 |
|  | Passeridae | *Passer domesticus* | 0 | 0 | 0 | 0 | 1 | 0 |
|  | Pipridae | *Antilophia galeata* | 0 | 0 | 0 | 0 | 2 | 1 |
|  | Polioptilidae | *Polioptila plumbea* | 3 | 7 | 0 | 0 | 0 | 0 |
|  | Rhynchocyclidae | *Hemithraupis guira* | 0 | 0 | 0 | 0 | 0 | 3 |
|  |  | *Hemitriccus margaritaceiventer* | 1 | 4 | 0 | 0 | 0 | 2 |
|  |  | *Todirostrum cinereum* | 3 | 0 | 0 | 0 | 2 | 0 |
|  |  | *Tolmomyias flaviventris* | 1 | 1 | 0 | 0 | 0 | 0 |
|  |  | *Tolmomyias sulphurescens* | 0 | 0 | 0 | 0 | 0 | 2 |
|  | Thamnophilidae | *Formicivora grisea* | 2 | 3 | 0 | 0 | 0 | 0 |
|  |  | *Forpus xanthopterygius* | 0 | 2 | 0 | 2 | 0 | 0 |
|  |  | *Thamnophilus caerulescens* | 0 | 0 | 0 | 4 | 0 | 0 |
|  |  | *Thamnophilus doliatus* | 0 | 0 | 2 | 1 | 0 | 0 |
|  |  | *Thamnophilus torquatus* | 0 | 0 | 1 | 0 | 0 | 0 |
|  | Thraupidae | *Coereba flaveola* | 18 | 31 | 0 | 0 | 2 | 7 |
|  |  | *Coryphospingus cucullatus* | 0 | 0 | 29 | 8 | 0 | 3 |
|  |  | *Coryphospingus pileatus* | 0 | 2 | 0 | 0 | 0 | 0 |
|  |  | *Dacnis cayana* | 0 | 3 | 0 | 0 | 0 | 1 |
|  |  | *Nemosia pileata* | 1 | 0 | 2 | 1 | 0 | 0 |
|  |  | *Neothraupis fasciata* | 0 | 0 | 0 | 0 | 10 | 4 |
|  |  | *Saltator maximus* | 0 | 0 | 0 | 0 | 0 | 2 |
|  |  | *Saltator similis* | 0 | 0 | 0 | 0 | 0 | 1 |
|  |  | *Schistochlamys melanopis* | 0 | 0 | 0 | 0 | 1 | 0 |
|  |  | *Schistochlamys ruficapillus* | 0 | 6 | 0 | 0 | 0 | 0 |
|  |  | *Sicalis flaveola* | 0 | 2 | 0 | 0 | 0 | 0 |
|  |  | *Sporophila caerulescens* | 0 | 0 | 0 | 0 | 0 | 1 |
|  |  | *Sporophila leucoptera* | 0 | 0 | 0 | 0 | 3 | 0 |
|  |  | *Sporophila nigricollis* | 0 | 0 | 0 | 0 | 1 | 1 |
|  |  | *Sporophila plumbea* | 0 | 0 | 0 | 0 | 0 | 2 |
|  |  | *Tachyphonus rufus* | 1 | 4 | 0 | 0 | 0 | 4 |
|  |  | *Tangara cayana* | 0 | 7 | 0 | 3 | 0 | 14 |
|  |  | *Thlypopsis sordida* | 1 | 1 | 1 | 0 | 0 | 0 |
|  |  | *Thraupis palmarum* | 1 | 5 | 0 | 0 | 0 | 0 |
|  |  | *Thraupis sayaca* | 21 | 8 | 10 | 14 | 1 | 0 |
|  |  | *Volatinia jacarina* | 0 | 0 | 39 | 4 | 57 | 24 |
|  | Tityridae | *Pachyramphus polychopterus* | 0 | 0 | 0 | 0 | 1 | 2 |
|  | Troglodytidae | *Troglodytes aedon* | 10 | 12 | 16 | 12 | 12 | 12 |
|  | Turdidae | *Turdus amaurochalinus* | 0 | 0 | 0 | 0 | 1 | 1 |
|  |  | *Turdus leucomelas* | 17 | 16 | 8 | 14 | 14 | 1 |
|  |  | *Turdus rufiventris* | 2 | 4 | 0 | 1 | 3 | 1 |
|  | Tyrannidae | *Camptostoma obsoletum* | 1 | 3 | 1 | 8 | 4 | 2 |
|  |  | *Cnemotriccus fuscatus* | 0 | 0 | 1 | 3 | 0 | 0 |
|  |  | *Elaenia chiriquensis* | 0 | 1 | 1 | 0 | 43 | 66 |
|  |  | *Elaenia cristata* | 0 | 8 | 0 | 0 | 18 | 17 |
|  |  | *Elaenia flavogaster* | 3 | 0 | 0 | 0 | 5 | 0 |
|  |  | *Elaenia mesoleuca* | 0 | 0 | 0 | 0 | 1 | 0 |
|  |  | *Elaenia obscura* | 0 | 0 | 0 | 0 | 1 | 0 |
|  |  | *Elaenia parvirostris* | 0 | 0 | 0 | 0 | 0 | 1 |
|  |  | *Empidonomus varius* | 0 | 0 | 0 | 1 | 1 | 0 |
|  |  | *Fluvicola nengeta* | 0 | 1 | 0 | 0 | 0 | 0 |
|  |  | *Lathrotriccus euleri* | 0 | 0 | 0 | 0 | 0 | 1 |
|  |  | *Megarynchus pitangua* | 0 | 1 | 0 | 0 | 0 | 0 |
|  |  | *Myiarchus swainsoni* | 0 | 1 | 2 | 5 | 8 | 17 |
|  |  | *Myiarchus tyrannulus* | 0 | 0 | 0 | 6 | 0 | 0 |
|  |  | *Myiodynastes maculatus* | 0 | 0 | 1 | 1 | 1 | 0 |
|  |  | *Myiophobus fasciatus* | 0 | 0 | 1 | 0 | 7 | 4 |
|  |  | *Myiozetetes similis* | 4 | 0 | 0 | 0 | 0 | 0 |
|  |  | *Phaeomyias murina* | 0 | 0 | 0 | 0 | 0 | 2 |
|  |  | *Pitangus sulphuratus* | 41 | 10 | 7 | 0 | 6 | 0 |
|  |  | *Serpophaga subcristata* | 0 | 0 | 1 | 0 | 0 | 0 |
|  |  | *Suiriri suiriri* | 0 | 0 | 0 | 0 | 3 | 4 |
|  |  | *Thryothorus leucotis* | 0 | 0 | 0 | 0 | 1 | 0 |
|  |  | *Tyrannus melancholicus* | 4 | 0 | 1 | 0 | 1 | 0 |
|  | Vireonidae | *Cyclarhis gujanensis* | 9 | 6 | 0 | 3 | 1 | 1 |
|  |  | *Hylophilus amaurocephalus* | 0 | 3 | 0 | 3 | 0 | 0 |
|  |  | *Vireo olivaceus* | 0 | 0 | 0 | 2 | 0 | 0 |
| Piciformes | Picidae | *Dryocopus lineatus* | 0 | 0 | 0 | 1 | 0 | 0 |
|  |  | *Picumnus pygmaeus* | 1 | 1 | 0 | 0 | 0 | 0 |
|  |  | *Veniliornis mixtus* | 0 | 0 | 0 | 0 | 0 | 1 |
|  |  | *Veniliornis passerinus* | 0 | 0 | 0 | 0 | 1 | 0 |
| Strigiformes | Strigidae | *Megascops choliba* | 0 | 0 | 0 | 1 | 0 | 0 |

Supplemental Table 2: Species identity, total number of captures, number of individuals infected by haemosporidian parasites and prevalence (number of infected divided by the number of captured individuals).

| Species | Total | Infected | Prevalence |
| --- | --- | --- | --- |
| *Ammodramus humeralis* | 9 | 0 | 0 |
| *Antilophia galeata* | 3 | 0 | 0 |
| *Arremon flavirostris* | 1 | 0 | 0 |
| *Basileuterus culicivorus* | 1 | 0 | 0 |
| *Basileuterus leucophrys* | 1 | 0 | 0 |
| *Buteo magnirostris* | 1 | 0 | 0 |
| *Camptostoma obsoletum* | 19 | 1 | 5.26 |
| *Caprimulgus maculicaudus* | 3 | 2 | 66.67 |
| *Caprimulgus parvulus* | 2 | 0 | 0 |
| *Cnemotriccus fuscatus* | 4 | 1 | 25 |
| *Coereba flaveola* | 58 | 6 | 10.34 |
| *Columbina minuta* | 1 | 0 | 0 |
| *Columbina passerina* | 3 | 2 | 66.67 |
| *Columbina squammata* | 1 | 0 | 0 |
| *Columbina talpacoti* | 9 | 1 | 11.11 |
| *Coryphospingus cucullatus* | 40 | 8 | 20 |
| *Coryphospingus pileatus* | 2 | 1 | 50 |
| *Crotophaga ani* | 11 | 1 | 9.09 |
| *Cyclarhis gujanensis* | 20 | 1 | 5 |
| *Dacnis cayana* | 4 | 1 | 25 |
| *Dryocopus lineatus* | 1 | 1 | 100 |
| *Elaenia chiriquensis* | 111 | 19 | 17.12 |
| *Elaenia cristata* | 43 | 13 | 30.23 |
| *Elaenia flavogaster* | 8 | 0 | 0 |
| *Elaenia mesoleuca* | 1 | 0 | 0 |
| *Elaenia obscura* | 1 | 0 | 0 |
| *Elaenia parvirostris* | 1 | 0 | 0 |
| *Empidonomus varius* | 2 | 0 | 0 |
| *Euphonia chlorotica* | 2 | 0 | 0 |
| *Fluvicola nengeta* | 1 | 0 | 0 |
| *Formicivora grisea* | 5 | 2 | 40 |
| *Forpus xanthopterygius* | 4 | 3 | 75 |
| *Furnarius rufus* | 5 | 1 | 20 |
| *Galbula ruficauda* | 1 | 0 | 0 |
| *Geothlypis velata* | 1 | 0 | 0 |
| *Hemithraupis guira* | 3 | 0 | 0 |
| *Hemitriccus margaritaceiventer* | 7 | 1 | 14.29 |
| *Hylophilus amaurocephalus* | 6 | 2 | 33.33 |
| *Icterus cayanensis* | 1 | 0 | 0 |
| *Lathrotriccus euleri* | 1 | 0 | 0 |
| *Lepidocolaptes angustirostris* | 8 | 1 | 12.5 |
| *Leptotila rufaxilla* | 1 | 0 | 0 |
| *Leptotila verreauxi* | 2 | 1 | 50 |
| *Megarynchus pitangua* | 1 | 0 | 0 |
| *Megascops choliba* | 1 | 1 | 100 |
| *Mimus gilvus* | 7 | 2 | 28.57 |
| *Mimus saturninus* | 1 | 1 | 100 |
| *Myiarchus swainsoni* | 33 | 11 | 33.33 |
| *Myiarchus tyrannulus* | 6 | 0 | 0 |
| *Myiodynastes maculatus* | 3 | 0 | 0 |
| *Myiophobus fasciatus* | 12 | 1 | 8.33 |
| *Myiozetetes similis* | 4 | 1 | 25 |
| *Nemosia pileata* | 4 | 0 | 0 |
| *Neothraupis fasciata* | 14 | 10 | 71.43 |
| *Nyctidromus albicollis* | 2 | 1 | 50 |
| *Nystalus maculatus* | 2 | 0 | 0 |
| *Pachyramphus polychopterus* | 3 | 1 | 33.33 |
| *Passer domesticus* | 1 | 0 | 0 |
| *Phaeomyias murina* | 2 | 1 | 50 |
| *Picumnus pygmaeus* | 2 | 1 | 50 |
| *Pitangus sulphuratus* | 64 | 1 | 1.56 |
| *Polioptila plumbea* | 10 | 0 | 0 |
| *Pseudoseisura cristata* | 1 | 0 | 0 |
| *Saltator maximus* | 2 | 0 | 0 |
| *Saltator similis* | 1 | 0 | 0 |
| *Schistochlamys melanopis* | 1 | 0 | 0 |
| *Schistochlamys ruficapillus* | 6 | 1 | 16.67 |
| *Serpophaga subcristata* | 1 | 0 | 0 |
| *Sicalis flaveola* | 2 | 1 | 50 |
| *Sporophila caerulescens* | 1 | 0 | 0 |
| *Sporophila leucoptera* | 3 | 2 | 66.67 |
| *Sporophila nigricollis* | 2 | 0 | 0 |
| *Sporophila plumbea* | 2 | 0 | 0 |
| *Stelgidopteryx ruficollis* | 2 | 0 | 0 |
| *Suiriri suiriri* | 7 | 4 | 57.14 |
| *Synallaxis albescens* | 6 | 2 | 33.33 |
| *Synallaxis frontalis* | 22 | 4 | 18.18 |
| *Synallaxis spixi* | 2 | 0 | 0 |
| *Tachyphonus rufus* | 9 | 1 | 11.11 |
| *Tangara cayana* | 24 | 9 | 37.5 |
| *Thamnophilus caerulescens* | 4 | 4 | 100 |
| *Thamnophilus doliatus* | 3 | 0 | 0 |
| *Thamnophilus torquatus* | 1 | 0 | 0 |
| *Thlypopsis sordida* | 3 | 0 | 0 |
| *Thraupis palmarum* | 6 | 0 | 0 |
| *Thraupis sayaca* | 54 | 15 | 27.78 |
| *Thryothorus leucotis* | 1 | 0 | 0 |
| *Todirostrum cinereum* | 5 | 0 | 0 |
| *Tolmomyias flaviventris* | 2 | 1 | 50 |
| *Tolmomyias sulphurescens* | 2 | 0 | 0 |
| *Troglodytes aedon* | 74 | 14 | 18.92 |
| *Turdus amaurochalinus* | 2 | 0 | 0 |
| *Turdus leucomelas* | 70 | 31 | 44.29 |
| *Turdus rufiventris* | 11 | 2 | 18.18 |
| *Tyrannus melancholicus* | 6 | 0 | 0 |
| *Veniliornis mixtus* | 1 | 0 | 0 |
| *Veniliornis passerinus* | 1 | 0 | 0 |
| *Vireo olivaceus* | 2 | 0 | 0 |
| *Volatinia jacarina* | 124 | 26 | 20.97 |
| *Zonotrichia capensis* | 49 | 22 | 44.9 |

Supplemental Table 3: Host species identity, and the number of ectoparasites found in birds from airport and control locations.

| Species | Airport | Control |
| --- | --- | --- |
| *Ammodramus humeralis* | 0 | 1 |
| *Antilophia galeata* | 0 | 0 |
| *Arremon flavirostris* | 0 | 0 |
| *Basileuterus culicivorus* | 0 | 0 |
| *Basileuterus leucophrys* | 0 | 0 |
| *Buteo magnirostris* | 0 | 0 |
| *Camptostoma obsoletum* | 0 | 0 |
| *Caprimulgus maculicaudus* | 0 | 0 |
| *Caprimulgus parvulus* | 0 | 0 |
| *Cnemotriccus fuscatus* | 0 | 2 |
| *Coereba flaveola* | 1 | 1 |
| *Columbina minuta* | 0 | 0 |
| *Columbina passerina* | 0 | 0 |
| *Columbina squammata* | 0 | 0 |
| *Columbina talpacoti* | 0 | 0 |
| *Coryphospingus cucullatus* | 2 | 2 |
| *Coryphospingus pileatus* | 0 | 0 |
| *Crotophaga ani* | 0 | 0 |
| *Cyclarhis gujanensis* | 1 | 0 |
| *Dacnis cayana* | 0 | 0 |
| *Dryocopus lineatus* | 0 | 0 |
| *Elaenia chiriquensis* | 2 | 17 |
| *Elaenia cristata* | 2 | 0 |
| *Elaenia flavogaster* | 0 | 0 |
| *Elaenia mesoleuca* | 0 | 0 |
| *Elaenia obscura* | 0 | 0 |
| *Elaenia parvirostris* | 0 | 0 |
| *Empidonomus varius* | 0 | 0 |
| *Euphonia chlorotica* | 0 | 0 |
| *Fluvicola nengeta* | 0 | 0 |
| *Formicivora grisea* | 0 | 1 |
| *Forpus xanthopterygius* | 0 | 0 |
| *Furnarius rufus* | 0 | 0 |
| *Galbula ruficauda* | 0 | 0 |
| *Geothlypis velata* | 0 | 0 |
| *Hemithraupis guira* | 0 | 2 |
| *Hemitriccus margaritaceiventer* | 0 | 1 |
| *Hylophilus amaurocephalus* | 0 | 0 |
| *Icterus cayanensis* | 0 | 0 |
| *Lathrotriccus euleri* | 0 | 0 |
| *Lepidocolaptes angustirostris* | 0 | 1 |
| *Leptotila rufaxilla* | 0 | 0 |
| *Leptotila verreauxi* | 1 | 0 |
| *Megarynchus pitangua* | 0 | 0 |
| *Megascops choliba* | 0 | 0 |
| *Mimus gilvus* | 0 | 0 |
| *Mimus saturninus* | 0 | 0 |
| *Myiarchus swainsoni* | 0 | 3 |
| *Myiarchus tyrannulus* | 0 | 0 |
| *Myiodynastes maculatus* | 0 | 0 |
| *Myiophobus fasciatus* | 0 | 0 |
| *Myiozetetes similis* | 0 | 0 |
| *Nemosia pileata* | 0 | 0 |
| *Neothraupis fasciata* | 0 | 2 |
| *Nyctidromus albicollis* | 0 | 1 |
| *Nystalus maculatus* | 0 | 0 |
| *Pachyramphus polychopterus* | 0 | 0 |
| *Passer domesticus* | 0 | 0 |
| *Phaeomyias murina* | 0 | 0 |
| *Picumnus pygmaeus* | 0 | 0 |
| *Pitangus sulphuratus* | 4 | 0 |
| *Polioptila plumbea* | 0 | 0 |
| *Pseudoseisura cristata* | 0 | 0 |
| *Saltator maximus* | 0 | 1 |
| *Saltator similis* | 0 | 1 |
| *Schistochlamys melanopis* | 0 | 0 |
| *Schistochlamys ruficapillus* | 0 | 0 |
| *Serpophaga subcristata* | 0 | 0 |
| *Sicalis flaveola* | 0 | 0 |
| *Sporophila caerulescens* | 0 | 0 |
| *Sporophila leucoptera* | 0 | 0 |
| *Sporophila nigricollis* | 1 | 0 |
| *Sporophila plumbea* | 0 | 0 |
| *Stelgidopteryx ruficollis* | 0 | 0 |
| *Suiriri suiriri* | 0 | 0 |
| *Synallaxis albescens* | 0 | 0 |
| *Synallaxis frontalis* | 1 | 2 |
| *Synallaxis spixi* | 1 | 0 |
| *Tachyphonus rufus* | 0 | 0 |
| *Tangara cayana* | 0 | 4 |
| *Thamnophilus caerulescens* | 0 | 2 |
| *Thamnophilus doliatus* | 0 | 0 |
| *Thamnophilus torquatus* | 0 | 0 |
| *Thlypopsis sordida* | 0 | 0 |
| *Thraupis palmarum* | 0 | 0 |
| *Thraupis sayaca* | 0 | 0 |
| *Thryothorus leucotis* | 0 | 0 |
| *Todirostrum cinereum* | 0 | 0 |
| *Tolmomyias flaviventris* | 0 | 0 |
| *Tolmomyias sulphurescens* | 0 | 0 |
| *Troglodytes aedon* | 1 | 8 |
| *Turdus amaurochalinus* | 0 | 0 |
| *Turdus leucomelas* | 2 | 5 |
| *Turdus rufiventris* | 1 | 0 |
| *Tyrannus melancholicus* | 0 | 0 |
| *Veniliornis mixtus* | 0 | 0 |
| *Veniliornis passerinus* | 0 | 0 |
| *Vireo olivaceus* | 0 | 1 |
| *Volatinia jacarina* | 7 | 6 |
| *Zonotrichia capensis* | 0 | 10 |

Supplementary Table 4: Genus, lineage ID, bird host, and the number of haemosporidian-infected individuals / total number of sampled individuals within airports and control areas.

| Genus | Lineage ID | Bird host | Airport | Control |
| --- | --- | --- | --- | --- |
| *Haemoproteus* spp. | ZEMAC03 | *Columbina passerina* | 0/0 | 1/3 (33) |
| *Haemoproteus* *Parahaemoproteus* spp. | EMPOBE02 | *Elaenia cristata* | 0/18 | 1/25 (4) |
|  | CAPMAC01 | *Caprimulgus maculicaudus* | 1/2 | 0/1 |
|  | NEOFAS02 | *Neothraupis fasciata* | 1/10 | 0/4 |
|  |  | *Thraupis sayaca* | 2/32 | 0/22 |
|  | NEOFAS03 | *Elaenia cristata* | 1/18 | 0/25 |
|  |  | *Elaenia chiriquensis* | 0/44 | 1/67 |
|  |  | *Neothraupis fasciata* | 4/10 | 1/4 |
|  | SUISUI02 | *Suiriri suiriri* | 1/3 | 0/4 |
|  | UN139 | *Zonotrichia capensis* | 0/8 | 1/41 |
|  | VIOLI06 | *Cyclarhis gujanensis* | 1/10 | 0/10 |
| *Plasmodium* spp. | VOLJAC02 | *Elaenia chiriquensis* | 0/44 | 1/67 |
|  |  | *Zonotrichia capensis* | 0/8 | 1/41 |
|  | BAHYP01 | *Sporophila leucoptera* | 1/3 | 0/0 |
|  |  | *Volatinia jacarina* | 1/96 | 0/28 |
|  | DENPET03 | *Myiarchus swainsoni* | 1/10 | 0/23 |
|  |  | *Tangara cayana* | 0/0 | 1/24 |
|  |  | *Volatinia jacarina* | 1/96 | 0/28 |
|  |  | *Zonotrichia capensis* | 0/8 | 1/41 |
|  | MOLATE03 | *Neothraupis fasciata* | 0/10 | 1/4 |
|  | MASTR01 | *Turdus leucomelas* | 0/39 | 4/31 |
|  | MIMSAT01 | *Mimus saturninus* | 1/1 | 0/0 |
|  | PADOM09 | *Myiarchus swainsoni* | 1/10 | 0/23 |
|  |  | *Troglodytes aedon* | 2/38 | 0/36 |
|  | PADOM11 | *Coryphospingus cucullatus* | 1/29 | 0/11 |
|  |  | *Neothraupis fasciata* | 2/10 | 0/4 |
|  |  | *Volatinia jacarina* | 1/96 | 0/28 |
|  | PSABIF01 | *Megascops choliba* | 0/0 | 1/1 |
|  | RAMCAR01 | *Elaenia chiriquensis* | 1/44 | 0/67 |
|  | SPMAG11 | *Thraupis sayaca* | 1/32 | 0/22 |
|  | TARUF01 | *Neothraupis fasciata* | 1/10 | 0/4 |
|  | TULEU06 | *Turdus leucomelas* | 1/39 | 6/31 |
|  | TULEU07 | *Turdus leucomelas* | 0/39 | 1/31 |
|  | TUMIG03 | *Turdus rufiventris* | 1/5 | 0/6 |
|  | RHYSIM01 | *Elaenia chiriquensis* | 2/44 | 0/67 |
|  |  | *Turdus leucomelas* | 1/39 | 1/31 |
|  |  | *Turdus rufiventris* | 0/5 | 1/6 |

Supplementary Table 5: Family, bird hosts and the quantity of ectoparasite-infested hosts in the airports and control areas.

| Family | Bird hosts | Airport | Control |
| --- | --- | --- | --- |
| Hippoboscidae | *Leptotila verreauxi* | 1/1 | 0/1 |
|  | *Pitangus sulphuratus* | 1/54 | 0/10 |
|  | *Synallaxis frontalis* | 0/16 | 1/6 |
|  | *Sporophila nigricollis* | 1/1 | 0/1 |
| Ixodidae | *Ammodramus humeralis* | 1/4 | 0/5 |
|  | *Cnemotriccus fuscatus* | 0/1 | 1/3 |
|  | *Coereba flaveola* | 0/20 | 1/38 |
|  | *Coryphospingus cucullatus* | 0/29 | 1/11 |
|  | *Elaenia chiriquensis* | 2/44 | 2/67 |
|  | *Elaenia cristata* | 0/18 | 2/25 |
|  | *Hemithraupis guira* | 0/0 | 1/3 |
|  | *Lepidocolaptes angustirostris* | 1/2 | 0/6 |
|  | *Leptotila verreauxi* | 1/1 | 0/1 |
|  | *Myiarchus swainsoni* | 0/10 | 2/23 |
|  | *Neothraupis fasciata* | 1/10 | 0/4 |
|  | *Saltator maximus* | 0/0 | 1/2 |
|  | *Saltator similis* | 0/0 | 1/1 |
|  | *Synallaxis frontalis* | 1/16 | 2/6 |
|  | *Tangara cayana* | 2/0 | 0/24 |
|  | *Thamnophilus caerulescens* | 0/0 | 2/4 |
|  | *Troglodytes aedon* | 0/38 | 3/36 |
|  | *Turdus leucomelas* | 2/39 | 0/31 |
|  | *Vireo olivaceus* | 0/0 | 1/2 |
|  | *Volatinia jacarina* | 0/96 | 4/28 |
|  | *Zonotrichia capensis* | 1/8 | 0/41 |
| Trombiculidae | *Coryphospingus cucullatus* | 0/29 | 2/11 |
|  | *Cyclarhis gujanensis* | 1/10 | 0/10 |
|  | *Elaenia chiriquensis* | 1/44 | 15/67 |
|  | *Formicivora grisea* | 0/2 | 1/3 |
|  | *Hemithraupis guira* | 0/0 | 1/3 |
|  | *Myiarchus swainsoni* | 0/10 | 3/23 |
|  | *Nyctidromus albicollis* | 0/0 | 1/2 |
|  | *Pitangus sulphuratus* | 3/54 | 0/10 |
|  | *Synallaxis spixi* | 1/1 | 0/1 |
|  | *Tangara cayana* | 0/0 | 2/24 |
|  | *Troglodytes aedon* | 0/38 | 8/36 |
|  | *Turdus leucomelas* | 2/39 | 3/31 |
|  | *Turdus rufiventris* | 1/5 | 0/6 |
|  | *Volatinia jacarina* | 3/96 | 6/28 |
|  | *Zonotrichia capensis* | 0/8 | 8/41 |

Supplementary Table 6: Parasite coinfections according to bird species and location.

| Bird host | N | Haemosporida | Ectoparasite | Location |
| --- | --- | --- | --- | --- |
| *Coryphospingus cucullatus* | 2/29 | NI | Ixodidae | Campinas airport |
| *Formicivora grisea* | 1/2 | NI | Trombiculidae | Salvador control |
| *Leptotila verreauxi* | 1/1 | NI | Hippoboscidae | Campinas airport |
| *Nyctidromus albicollis* | 1/2 | NI | Trombiculidae | Salvador control |
| *Pitangus sulphuratus* | 1/7 | NI | Hippoboscidae | Campinas airport |
| *Tangara cayana* | 1/14 | NI | Trombiculidae | Brasilia control |
| *Thamnophilus caerulescens* | 2/4 | NI | Ixodidae | Campinas control |
| *Turdus leucomelas* | 3/16 | NI | Trombiculidae | Salvador control |
|  | 1/14 | MASTR01 | Ixodidae | Campinas control |
|  | 1/14 | TULEU06 | Ixodidae | Campinas control |
| *Turdus rufiventris* | 1/3 | TUMIG03 | Trombiculidae | Brasilia airport |

Bird species name, number of co-infected individuals (N) / total sample size, and the co-occurrence of haemosporidian parasites (name of lineage when possible), ectoparasite (family’s name), and the location where the birds were captured are displayed.NI - Haemosporida not identified to genus level.

Supplementary Table 7: Summary of Haemosporidian Lineages Detected Across Cities and Site Types, Showing Lineage Identity and Frequency of Infected Hosts. Number of Unique Haemosporidian Lineages Detected by Site Type: Brasília (Airport = 17, Control = 7), Campinas (Airport = 5, Control = 7), and Salvador (Control = 3)

| City | Location | Lineage | Sample size |
| --- | --- | --- | --- |
| Brasilia | Airport | NEOFAS03 | 5 |
| Brasilia | Airport | PADOM11 | 3 |
| Brasilia | Airport | TURUF01 | 3 |
| Brasilia | Airport | DENPET03 | 3 |
| Brasilia | Airport | NEOFAS02 | 2 |
| Brasilia | Airport | BAHYP01 | 1 |
| Brasilia | Airport | BAHYP01 | 1 |
| Brasilia | Airport | *Haemoproteus virenionis* | 1 |
| Brasilia | Airport | PRAIRIECH | 1 |
| Brasilia | Airport | *Plasmodium cathemerium* | 1 |
| Brasilia | Airport | RAMCAR 01 | 1 |
| Brasilia | Airport | SUISUI02 | 1 |
| Brasilia | Airport | TARUF01 | 1 |
| Brasilia | Airport | TULEU06 | 1 |
| Brasilia | Airport | TUMIG03 | 1 |
| Brasilia | Control | DENPET03 | 3 |
| Brasilia | Control | BAFLA03 | 2 |
| Brasilia | Control | NEOFAS03 | 2 |
| Brasilia | Control | EMPOBE02 | 1 |
| Brasilia | Control | IIL-2016 | 1 |
| Brasilia | Control | Mix (SUISUI02 e MYMAC02) | 1 |
| Campinas | Airport | PADOM09 | 3 |
| Campinas | Airport | NEOFAS02 | 1 |
| Campinas | Airport | PADOM11 | 1 |
| Campinas | Airport | STTA17H | 1 |
| Campinas | Control | TULEU06 | 6 |
| Campinas | Control | *Haemoproteus erythrogravidus* | 5 |
| Campinas | Control | MASTR01 | 2 |
| Campinas | Control | PsDIS01 | 1 |
| Campinas | Control | TULEU07 | 1 |
| Campinas | Control | TURUF01 | 1 |
| Campinas | Control | ZC2 | 1 |
| Salvador | Control | MASTR01 | 1 |
| Salvador | Control | Mix (TURUF01 e MASTR01) | 1 |
| Salvador | Control | SocH3 | 1 |

Supplementary Table 8: R² Values from Per-Species, Per-Site Linear Regressions of Body Mass on Tarsus Length Used to Estimate Body Condition Residuals

| Species | Site | R2 |
| --- | --- | --- |
| *Elaenia chiriquensis* | Brasilia Airport | 0.067 |
| *Elaenia chiriquensis* | Brasilia Control | 0.075 |
| *Myiarchus swainsoni* | Brasilia Airport | 0.15 |
| *Myiarchus swainsoni* | Campinas Control | 0.301 |
| *Myiarchus swainsoni* | Brasilia Control | 0.138 |
| *Camptostoma obsoletum* | Campinas Control | 0.091 |
| *Neothraupis fasciata* | Brasilia Airport | 0.263 |
| *Elaenia cristata* | Brasilia Airport | 0.521 |
| *Elaenia cristata* | Salvador Control | 0.005 |
| *Elaenia cristata* | Brasilia Control | 0.411 |
| *Lepidocolaptes angustirostris* | Brasilia Control | 0.167 |
| *Myiophobus fasciatus* | Brasilia Airport | 0.016 |
| *Elaenia flavogaster* | Brasilia Airport | 0.049 |
| *Volatinia jacarina* | Brasilia Airport | 0.02 |
| *Volatinia jacarina* | Campinas Airport | 0.065 |
| *Volatinia jacarina* | Brasilia Control | 0.069 |
| *Troglodytes aedon* | Brasilia Airport | 0.001 |
| *Troglodytes aedon* | Salvador Airport | 0.012 |
| *Troglodytes aedon* | Campinas Airport | 0.068 |
| *Troglodytes aedon* | Salvador Control | 0.205 |
| *Troglodytes aedon* | Campinas Control | 0.004 |
| *Troglodytes aedon* | Brasilia Control | 0.389 |
| *Crotophaga ani* | Brasilia Airport | 0.012 |
| *Crotophaga ani* | Salvador Airport | 0.356 |
| *Turdus leucomelas* | Brasilia Airport | 0.084 |
| *Turdus leucomelas* | Salvador Airport | 0.141 |
| *Turdus leucomelas* | Campinas Airport | 0.029 |
| *Turdus leucomelas* | Salvador Control | 0.197 |
| *Turdus leucomelas* | Campinas Control | 0.117 |
| *Cyclarhis gujanensis* | Salvador Airport | 0.104 |
| *Cyclarhis gujanensis* | Salvador Control | 0.426 |
| *Synallaxis frontalis* | Campinas Airport | 0.149 |
| *Zonotrichia capensis* | Campinas Airport | 0.006 |
| *Zonotrichia capensis* | Campinas Control | 0.085 |
| *Zonotrichia capensis* | Brasilia Control | 0.001 |
| *Pitangus sulphuratus* | Brasilia Airport | 0.189 |
| *Pitangus sulphuratus* | Salvador Airport | 0.359 |
| *Pitangus sulphuratus* | Campinas Airport | 0.005 |
| *Pitangus sulphuratus* | Salvador Control | 0.026 |
| *Thraupis sayaca* | Salvador Airport | 0.084 |
| *Thraupis sayaca* | Campinas Airport | 0.836 |
| *Thraupis sayaca* | Salvador Control | 0.099 |
| *Thraupis sayaca* | Campinas Control | 0.022 |
| *Coereba flaveola* | Salvador Airport | 0.04 |
| *Coereba flaveola* | Salvador Control | 0.047 |
| *Coereba flaveola* | Brasilia Control | 0.157 |
| *Polioptila plumbea* | Salvador Control | 0.303 |
| *Thraupis palmarum* | Salvador Control | 0.057 |
| *Coryphospingus cucullatus* | Campinas Airport | 0.039 |
| *Coryphospingus cucullatus* | Campinas Control | 0.002 |
| *Tangara cayana* | Salvador Control | 0.002 |
| *Tangara cayana* | Brasilia Control | 0.061 |
| *Schistochlamys ruficapillus* | Salvador Control | 0.023 |
| *Myiarchus tyrannulus* | Campinas Control | 0.102 |
| *Synallaxis albescens* | Brasilia Control | 0.488 |
